# Constant-Envelope Waveform Design and Phase Recovery for Integrated Sensing and Communication in High-Mobility Multipath Environments

**DOI:** 10.3390/s26165130

**Published:** 2026-08-13

**Authors:** Wenhui Xue, Peng Chen, Chunguo Li, Zhenxin Cao, Shuqin Zhang

**Affiliations:** 1 School of Information Science and Engineering, Southeast University, Nanjing 210096, China; xuewenhui@seu.edu.cn; 2 Jiang Nan Design & Research Institute of Machinery & Electricity, Guiyang 550000, China; 220231062@seu.edu.cn; 3 State Key Laboratory of Millimeter Waves, Southeast University, Nanjing 210096, China; caozx@seu.edu.cn

**Keywords:** integrated sensing and communication, dual-functional radar–communication, orthogonal time frequency space, continuous phase modulation, constant-envelope waveform, multipath equalization

## Abstract

High-mobility dual-functional radar–communication systems require a common waveform that combines delay–Doppler information organization, sensing resolution, and power-efficient transmission. We present a cyclically closed constant-envelope orthogonal time frequency space–continuous phase modulation–linear frequency modulation (OTFS–CPM–LFM) waveform and matched transceiver architecture. Hermitian delay–Doppler mapping and direct-current (DC) row nulling create a real, zero-sum drive with a reversible frame-level phase representation. The communication receiver combines a Tikhonov-regularized waveform inverse with reference-aided unwrapping and tail-biting phase regression, while the radar receiver reconstructs the data-dependent current-frame reference. Numerical results verify the structural waveform properties and characterize communication, radar, and computational tradeoffs. They also quantify degradation under controlled complex-gain channel-state-information mismatch and show that phase regression is less reliable at a low signal-to-noise ratio (SNR). The constant-envelope claim applies only to ideal discrete complex-baseband samples and does not include pulse shaping or radio-frequency hardware. The framework therefore provides a self-consistent waveform interface while exposing tradeoffs among payload, recovery reliability, sensing sidelobes, and implementation cost.

## 1. Introduction

Integrated sensing and communication (ISAC) reuses spectrum, antenna aperture, radio-frequency hardware, and baseband resources to convey information while acquiring environmental state [1,2,3,4,5]. A common waveform must carry data and remain reconstructible as a sensing reference, coupling communication rate, sensing bounds, and transmit-signal structure [6]. High-speed aircraft, unmanned platforms, vehicular links, and low-altitude networks add pronounced Doppler, time-varying multipath, and restricted peak-power headroom. Studies of sensing-assisted beam prediction, multi-unmanned-aerial-vehicle resource allocation, and reconfigurable-intelligent-surface-assisted vehicular direction finding illustrate the coordination demands of dynamic ISAC networks [7,8,9]. Low-altitude intelligent-network and space–air–ground studies extend this context to joint security, beam, spectrum, and trajectory management under obstacles and resource constraints [10,11]. These applications motivate power-efficient waveform design for high-mobility links. The present work addresses this waveform-level interface; deployment additionally requires synchronization, channel tracking, clutter suppression, pulse shaping, and hardware validation.

Orthogonal time frequency space (OTFS) modulation organizes information symbols in the delay–Doppler (DD) domain and spreads them across the time–frequency plane through a two-dimensional transform, providing a structured representation of time-varying channels [12,13,14,15,16,17]. Existing channel-estimation methods exploit three-dimensional sparsity, embedded DD-domain pilots, time–frequency-domain pilots, or low-peak-to-average-power-ratio (PAPR) superimposed pilots [18,19,20,21]. The relative performance of OTFS and orthogonal frequency-division multiplexing (OFDM) depends on channel sparsity, pulse shaping, and receiver processing [22]. In this work, the principal value of the DD domain is its finite-dimensional organization of independent information in high-mobility multipath channels. Conventional OTFS, however, carries complex DD symbols through a linear complex envelope and does not naturally provide constant-envelope transmission.

Several OTFS-based ISAC studies have demonstrated joint radar parameter estimation and communication, sensing-assisted vehicular transmission, and preprocessing for the industrial Internet of Things [12,23,24]. Superimposed training and learning-aided reception have also improved robustness to nonideal conditions [25]. An OTFS–chirp design introduces a swept-frequency structure to combine DD-domain organization with chirp correlation processing [26]. These representative transceivers remain primarily based on linear complex-valued OTFS waveforms. They address DD-domain estimation, detection, or chirp integration, but do not provide a reversible mapping from independent complex DD information to a real continuous-phase drive or enforce closure of the accumulated information phase at finite-frame boundaries.

In parallel, radar-centric approaches start from the time–bandwidth product and range-compression capability of linear frequency modulation (LFM) and embed communication information in phase, frequency, or index dimensions. LFM phase-shift keying (PSK), minimum-shift-keying (MSK)–LFM, and LFM binary-phase-shift-keyed multibeam systems demonstrate discrete-phase embedding, continuous-phase modulation, and array-beam sharing, respectively [27,28,29]. Energy-leakage analysis further shows that communication modulation changes radar correlation outputs and sidelobe structure [30]. These approaches retain an explicit wideband sensing backbone, but usually embed communication information symbol by symbol or segment by segment and do not directly connect it to frame-level DD-domain organization in high-mobility channels.

High PAPR in communication-centric multicarrier waveforms increases the linear backoff required by power amplifiers (PAs) [31]. Consequently, dual-functional radar–communication (DFRC) research has addressed constant-modulus or constrained-envelope optimization through symbol-level precoding, joint transmit beamforming, reconfigurable-intelligent-surface-assisted waveform design, secure transmission, and angular estimation [32,33,34,35,36]. Other studies impose PAPR constraints on multiple-input multiple-output (MIMO) DFRC or MIMO orthogonal frequency-division multiplexing waveforms, sometimes with sidelobe and spectrum constraints [37,38,39,40]. Quantized constant-envelope, constant-modulus orthogonal, and clutter-aware designs provide discrete-waveform or array-codeword solutions [41,42,43]. Their optimization variables differ from an explicit generative map among independent DD bits, a real frequency drive, and accumulated phase.

Continuous-phase modulation (CPM) produces a constant-envelope signal through the exponential mapping of a real-valued frequency drive and its accumulated phase [44], whereas conventional OTFS linearly maps complex DD symbols to a complex time-domain envelope. The two schemes differ in drive variables, information mapping, and receiver inversion. Clipping a conventional OTFS waveform, taking only its real part, or appending an extra phase modulation cannot simultaneously ensure DD-data invertibility, phase continuity, and frame-boundary consistency. This interface mismatch also precludes direct receiver reuse: linear OTFS interference cancellation, iterative detection, and channel-estimation methods [14,15,16,17,18,19,20,21] cannot bypass the phase-exponential mapping and operate directly on DD information. Once the real drive has been exponentiated and then superposed by a time-varying multipath channel, multipath summation, phase extraction, and samplewise differencing do not commute. The receiver must first recover the multipath-distorted complex waveform, resolve phase ambiguity, and then exploit the frame-level generative relationship to recover DD information.

Table 1 compares the structural relationships among representative waveform routes. Complete transceiver baselines are evaluated under a common simulation grid in Section 6.4.

The representative routes in Table 1 do not jointly resolve four coupled interfaces. Independent DD information must first become a recoverable real CPM drive. Its accumulated phase must then close within a finite frame. After phase exponentiation and multipath superposition, communication recovery must invert propagation before phase extraction. Finally, the radar must match the data-dependent current frame rather than a bare sensing carrier. The proposed finite-frame construction connects these interfaces through one shared generative model. Its contributions are as follows:

1. A cyclically closed OTFS–CPM–LFM map converts independent DD bits into a real zero-sum drive and constant-envelope composite phase. The fixed loss of structural degrees of freedom makes frame closure explicit.
2. A layered communication receiver applies full-path Tikhonov waveform inversion before reference-aided unwrapping and tail-biting phase regression (TB-PR). This order follows the physical sequence of phase modulation and multipath propagation.
3. A data-dependent radar branch reconstructs the actual current frame. Same-grid references and metric-specific ablations then separate template mismatch from waveform, recovery, and payload tradeoffs.

The real OTFS drive is obtained from a complete frame of DD bits through a linear mapping and therefore constitutes frame-level generalized CPM. It reduces to strict MSK only when the drive is binary and the classical MSK modulation-index condition holds; the term OTFS–CPM–LFM is used throughout. Section 2 establishes the communication and radar models and formalizes the four interface problems. Section 3 develops the real drive, phase closure, and constant-envelope waveform. Section 4 presents the data-dependent radar and strong-multipath communication receivers. Section 5 defines the metrics and feasible parameter region. Section 6 reports waveform, radar, communication, and tradeoff results, and Section 7 concludes the paper.

Notation. Bold lowercase and uppercase symbols denote vectors and matrices, respectively. The symbols 0 and I denote the conformable zero vector and identity matrix. The imaginary unit is $j=\sqrt{-1}$. Superscripts ${\left(\cdot \right)}^{T}$, ${\left(\cdot \right)}^{H}$, and ${\left(\cdot \right)}^{*}$ denote transpose, Hermitian transpose, and complex conjugation, respectively. The operators |·| and ${∥\cdot ∥}_{2}$ denote magnitude and Euclidean norm. The operators Re{·}, Im{·}, and ∠(·) return the real part, imaginary part, and principal complex phase. The operator diag{·} forms a diagonal matrix, ⊙ denotes elementwise multiplication, and ${\left(\cdot \right)}_{L}$ denotes modulo-*L* indexing. The notation CN denotes a circularly symmetric complex Gaussian distribution. The indices *q*, *k*, *l*, and *p* identify a time sample, Doppler bin, delay bin, and propagation path, respectively. The constants *c* and $f_{c}$ denote propagation speed and carrier frequency. Communication delays and Dopplers are one-way quantities, whereas radar delay and Doppler are defined separately for the two-way sensing path. The operators argmin, sign(·), and unwrap(·) denote minimization, componentwise sign decision, and phase unwrapping, respectively, while O(·) denotes asymptotic computational order. A hat denotes an estimate, and $\overset{˘}{b}$ denotes the continuous pre-decision output of phase regression.

## 2. System Model and Problem Formulation

We consider a single-platform, single-antenna common-waveform DFRC system. The transmitting node shares baseband processing, frequency generation, power amplification, and antenna aperture and emits one OTFS–CPM–LFM frame. One portion returns to a colocated radar receiver after target scattering, while another reaches a remote communication receiver through direct and scattered paths. The radar branch knows the current communication data and can reconstruct the actual transmitted waveform; the communication branch must recover the unknown DD bits from a multipath observation.

**Figure 1:**
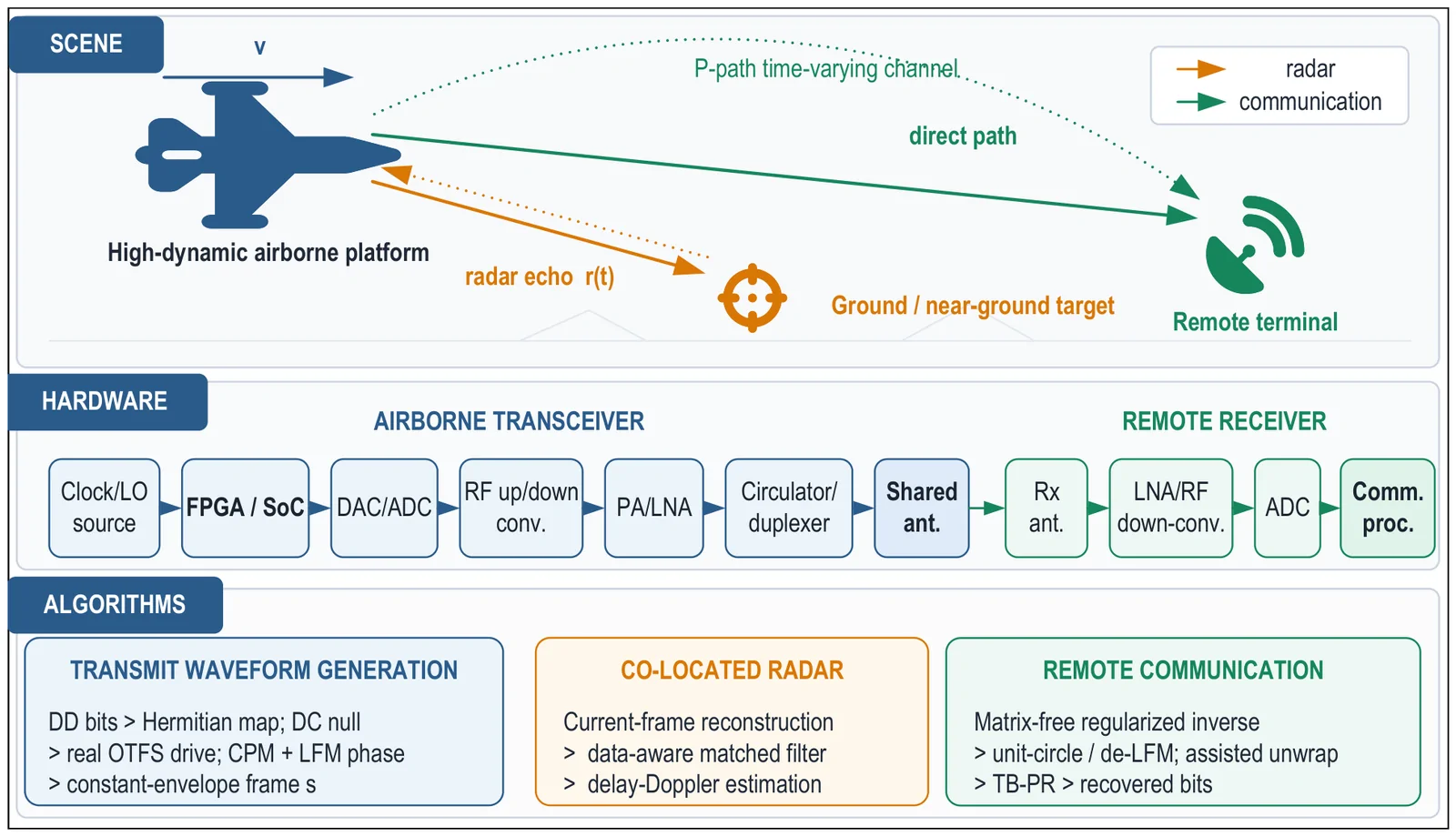
Application scenario and shared-aperture DFRC system architecture.

Figure 1 combines the application scene, hardware composition, and algorithm allocation. A high-mobility airborne platform, a ground or near-ground target, and a remote terminal are connected through a two-way radar link and a one-way time-varying multipath communication link. A shared-aperture airborne transceiver uses one antenna, a circulator or duplexer, and common radio-frequency and baseband resources; only the communication receiver chain resides at the remote terminal. A conceptual implementation partition places waveform generation and data-dependent radar processing on the onboard field-programmable gate array/system-on-chip, with matrix-free regularized inversion and phase recovery on the remote processor. The analysis focuses on the single-platform waveform and transceiver processing under perfect channel state information (CSI); array processing, beamforming, channel estimation, and multitarget processing are outside the system model.

**Figure 2:**
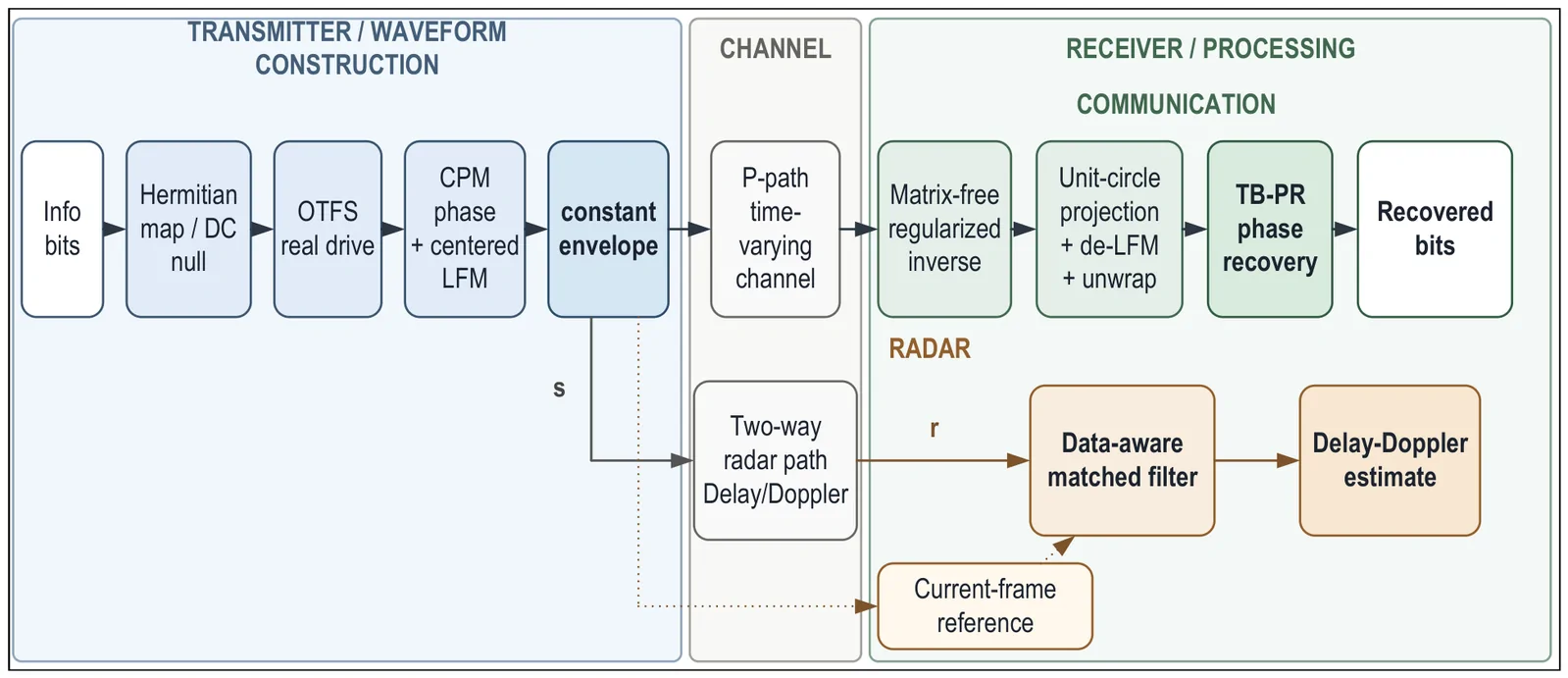
Frame-level processing flow of the proposed OTFS-CPM-LFM transceiver.

Figure 2 summarizes the frame-level signal flow. The transmitter performs Hermitian DD mapping and DC nulling, real-valued OTFS synthesis, CPM information-phase generation, centered LFM phase superposition, and phase exponentiation. The communication branch restores the complex waveform under a time-varying multipath model before phase preprocessing and TB-PR. The radar branch forms a data-dependent matched reference from the current-frame reconstruction and estimates delay and Doppler. Both branches share the actual transmitted frame without conflating one-way communication parameters with two-way radar parameters.

A single-frame, single-sweep discrete complex-baseband model is adopted: one OTFS frame aligns with one LFM sweep and contains *N* Doppler bins and *M* delay bins, yielding

L=NM.

The frame duration is $T_{f}$, the sampling rate is $f_{s}=L/T_{f}$, and $t_{q}=q/f_{s}$ for q=0,…,L−1; the subcarrier spacing is $\Delta f=f_{s}/M$. The DD symbol is X[k,l], with k∈{0,…,N−1} and l∈{0,…,M−1}. Rectangular pulses and critical sampling make the DD mapping, real-drive synthesis, and discrete-phase generation deterministic linear operators. In an oversampled pulse-shaped system, these operators must be reconstructed for the selected shaping filter.

After a guard interval at least as long as the maximum path delay has been removed, the communication block model with integer-sample delays and path-specific Doppler shifts is

(1) $$y=Hs+w,\;H=\sum_{p=0}^{P-1}h_{p}D\left(\nu_{p}\right)P_{c}\left(d_{p}\right),$$

where $h_{p}$, $d_{p}$, and $\nu_{p}$ are the complex gain, cyclic delay in samples, and one-way Doppler of path *p*, respectively, and $\tau_{p}=d_{p}/f_{s}$. The cyclic-shift matrix is $P_{c}\left(d_{p}\right)$ and

(2) $$D\left(\nu_{p}\right)=\mathrm{diag}{\left\{e^{j2\pi \nu_{p}q/f_{s}}\right\}}_{q=0}^{L-1}.$$

The noise is $w∼\mathrm{CN}(0,\sigma_{n}^{2}I)$. Under the narrowband Doppler approximation, the one-way radial velocity satisfies $\nu_{p}\approx v_{p}f_{c}/c$. Channel dynamics are described by $|\nu_{p}|/\Delta f$, the within-frame phase rotations $|\nu_{p}|T_{f}$, and interpath Doppler differences. To isolate waveform mapping and recovery, perfect CSI $\left\{h_{p},d_{p},\nu_{p}\right\}$ is assumed and $\sum_{p}{\left|h_{p}\right|}^{2}=1$. Equation (1) is the equivalent cyclic block channel after guard removal; guard, pilot, and coding overheads are discussed in Section 6.4.

For the colocated monostatic radar, delay and Doppler are two-way quantities and are distinct from $\tau_{p}$ and $\nu_{p}$. A single-target discrete echo is

(3) $$r=\alpha D\left(\nu_{r,0}\right)P_{z}\left(d_{r}\right)s+c+n_{r},$$

Here $P_{z}\left(d_{r}\right)$ is the zero-padded linear-delay operator over the *L*-sample radar observation, α is the complex target gain, c is clutter, and $n_{r}∼\mathrm{CN}\left(0,\sigma_{r}^{2}I\right)$. The sampled matched-filter search evaluates Equation (3) at the integer delay cell $\tau_{r,0}=d_{r}/f_{s}$ and Doppler cell $\nu_{r,0}$. The local physical parameters are written as $\tau_{r}=\tau_{r,0}+\delta \tau$ and $\nu_{r}=\nu_{r,0}+\delta \nu$, giving $R=c\tau_{r}/2$ and $v=\lambda \nu_{r}/2$, where $\lambda =c/f_{c}$. The conditional Cramér–Rao lower bound (CRLB) in Section 5 describes the continuous residual offsets about that aligned cell. The numerical radar analysis assumes a known complex target gain and a clutter-free single-target scene. Unknown-gain estimation and clutter suppression remain outside the model.

The transmit map G, communication recovery map $R_{c}$, and data-dependent radar map $R_{r}$ have the unified interfaces

$$\begin{array}{ccc} s & =G(b), & b\in {\left\{-1,+1\right\}}^{K},\\ \hat{b} & =R_{c}\left(y;H\right), & \\ \left({\hat{d}}_{r},{\hat{\nu }}_{r}\right) & =R_{r}\left(r;s\right). & \end{array}$$

The first problem is a physically consistent mapping from independent DD information to a constant-envelope waveform. Given $\left(N,M,f_{s},f_{d},B_{L}\right)$, the known bits b must be mapped to a real drive $u\in R^{L}$ and a complex frame s such that $\sum_{q}u\left[q\right]=0$, |s[q]|=1, the net accumulated information phase is zero over one frame, and instantaneous frequency remains within the discrete sampling boundary, while retaining K=(N−1)M recoverable independent bits. This is a reversible interface between complex DD information and a continuous-phase real frequency drive, not a truncation of a conventional complex OTFS waveform.

The second problem is nonlinear communication recovery under a strong multipath. The receiver observes y and, within this model, knows H, $\sigma_{n}^{2}$, and the waveform parameters; the unknowns are s and b. Because phase exponentiation precedes propagation, multipath complex-waveform superposition does not commute with phase extraction. Directly differencing y or directly applying a conventional linear OTFS detector to recover the DD bits is therefore not valid for the proposed nonlinear signal model. The complete multipath operator must first estimate the complex waveform, after which phase ambiguity is removed and the framewise phase relationship recovers the independent DD bits. This construction is intended to mitigate the observed high-signal-to-noise-ratio BER saturation of dominant-path differencing and the noise sensitivity of local phase differences.

The third problem is radar matching and parameter estimation with data modulation. The radar knows the current data and complete frame s, observes r, and estimates the two-way discrete delay and Doppler. Under the clutter-free conditional-CRLB model, α is known and c=0; unknown-gain estimation and clutter suppression are not addressed. The radar processor must construct a data-dependent reference from the actual composite-phase frame to avoid the mismatch of a bare-LFM reference.

The fourth problem is to delimit feasible waveform parameters and joint radar and communication tradeoffs. For fixed $T_{f}$, $f_{s}$, and candidate ranges, combinations of $\left(N,M,f_{d},B_{L}\right)$ must first satisfy real-drive, phase-closure, constant-envelope, and discrete-sampling constraints and are then evaluated by structural degrees of freedom, occupied bandwidth, bit error rate (BER), radar sidelobes, and receiver complexity. Rather than optimize a single global objective containing all metrics, this work constructs physically feasible transmit and receive maps and characterizes the sampled feasible region and multi-metric tradeoffs.

## 3. Constant-Envelope OTFS–CPM–LFM Waveform Construction

Let *N* be even. The valid DD grid is defined by

(4) $$\begin{array}{ccc} X[0,l] & =0, & \\ X[N-k,l] & =X^{*}\left[k,l\right], & k=1,\ldots ,N/2-1,\\ X[N/2,l] & \in \{-1,+1\}. & \end{array}$$

The positive-Doppler half-plane uses unit-energy quadrature phase-shift keying (QPSK), the Nyquist row uses binary phase-shift keying (BPSK), and the DC row carries no data. Thus, Equation (4) first specifies which DD entries are independent and then generates the negative-Doppler entries by conjugation; counting those independent coordinates gives

(5) $$K=2\left(\frac{N}{2}-1\right)M+M=\left(N-1\right)M,$$

while dividing this bit count by the 2NM real coordinates of an unconstrained quadrature grid gives the structural degree-of-freedom ratio

(6) $$\rho_{H}=\frac{N-1}{2N}.$$

Hermitian pairing is the step that makes the OTFS drive real; DC-row nulling is an additional closure constraint rather than a requirement for real-valued synthesis. If the real DC row also carried BPSK data, the Hermitian grid would provide NM=1024 independent bits. Nulling that row gives K=(N−1)M=960 bits and $\rho_{H}=0.46875$, so the closure cost is M=64 bits, or 1/N=6.25% of the real degrees of freedom available after Hermitian pairing. Taking the real part of an unconstrained complex OTFS frame would instead create a many-to-one information map unless the symbol labeling and receiver were redesigned; retaining a data-bearing DC row or subtracting a data-dependent mean would also require the displaced degree of freedom to be tracked, remapped, or terminated. The adopted zero-DC construction therefore pays an explicit, fixed payload cost in exchange for a linear one-to-one real-drive map with an exact zero sum for every legal frame.

Let $T_{\mathrm{OTFS}}\left\{\cdot \right\}$ denote the inverse symplectic finite Fourier transform–Heisenberg synthesis operator under rectangular critical sampling, with output flattened using q=nM+l. The unnormalized drive is

(7) $$x=T_{\mathrm{OTFS}}\left\{X\right\},$$

Hermitian symmetry makes the synthesis output real in exact arithmetic; Equation (8) then removes the fixed synthesis scale to obtain the normalized drive

(8) u=Re{x}L−1∥Re{x}∥22.

Every valid quadrature/binary grid has the same DD energy, and the adopted OTFS synthesis is energy-preserving up to a fixed scale. The normalization factor in Equation (8) is therefore identical for all legal binary-coordinate vectors. Consequently, normalization does not make the legal-frame mapping data-dependent. Stacking the independent real coordinates of Equation (4) in a fixed order gives $b\in {\left\{-1,+1\right\}}^{K}$ and

(9) $$u=Ab,\;A\in R^{L\times K}.$$

**Proposition** **1** (Real drive and tail biting)**.** *Under Equation* (4) and rectangular critical sampling, u *is real and satisfies*

(10) $$\sum_{q=0}^{L-1}u\left[q\right]=0.$$

**Proof.**  For fixed *l*, X[:,l] is Hermitian symmetric in the Doppler index; its inverse discrete Fourier transform is real, and the synthesized x[n,l] is therefore real. Summing over *n* retains only the Doppler DC coefficient X[0,l]. Since X[0,l]=0, every delay column has a zero time-domain sum, and summing over *l* yields Equation (10). Positive real normalization preserves both properties. □

Tail-biting closure makes each OTFS frame a self-contained phase block. Continuous CPM state propagation preserves the within-frame payload but couples each frame to an incoming phase state and allows state-estimation errors to propagate across frames. Explicit termination resets the state but consumes additional samples and changes the frame structure. Equation (10) instead enforces $\phi_{c}\left[L-1\right]=0$ for every data vector, so each subsequent frame starts from a deterministic information-phase state without a tail interval. This construction localizes phase-state uncertainty to one frame and aligns with the blockwise OTFS and equalization architecture at the fixed payload cost quantified above.

Let $f_{d}$ be the frequency coefficient controlling phase sensitivity. The first step converts this coefficient from hertz to radians per sample:

(11) $$\kappa =\frac{2\pi f_{d}}{f_{s}}.$$

The resulting radians-per-sample scale then weights the cumulative sum of the real drive to form the discrete CPM information phase:

(12) $$\phi_{c}\left[q\right]=\kappa \sum_{r=0}^{q}u\left[r\right].$$

Equation (10) gives $\phi_{c}\left[L-1\right]=0$. In an ideal continuous-time representation centered on the frame midpoint, the LFM phase is

(13) $$\phi_{L}\left(t\right)=\pi \mu {\left(t-T_{f}/2\right)}^{2},\;\mu =\frac{B_{L}}{T_{f}}.$$

Here, $B_{L}$ is the LFM sweep bandwidth and μ is its frequency slope. Because $\phi_{L}\left(0\right)=\phi_{L}\left(T_{f}\right)$, consecutive continuous-time sweep boundaries have equal LFM phases. The discrete implementation uses $t_{q}=q/f_{s}$ and its last sample is at $T_{f}-1/f_{s}$; endpoint closure refers to the continuous frame boundary $t=T_{f}$. The final common waveform is

(14) $$s\left[q\right]=\exp\left\{j\left(\phi_{L}\left[q\right]+\phi_{c}\left[q\right]\right)\right\}.$$

Thus, |s[q]|=1. With the discrete sample-power definition $\max_{q}{\left|s\left[q\right]\right|}^{2}/(L^{-1}\sum_{q}{\left|s\left[q\right]\right|}^{2})$, the PAPR is $10\log_{10}1=0$ dB. This property applies to ideal discrete complex-baseband samples. It does not establish a constant envelope after pulse shaping, digital-to-analog converter (DAC) reconstruction, analog filtering, PA distortion, or radio-frequency (RF) transmission. The generalized CPM drive u[q] is a continuous real value obtained from the complete DD frame through A, rather than a conventional finite-alphabet CPM state. Both nevertheless share continuous phase and constant envelope. For q=1,…,L−1, the exact backward phase increment is $\Delta \phi \left[q\right]=\left(2\pi /f_{s}\right)\left\{\mu \left(t_{q}-T_{f}/2-1/\left(2f_{s}\right)\right)+f_{d}u\left[q\right]\right\}$. The implemented sampling screen evaluates the corresponding continuous-time instantaneous frequency at the sample times $t_{q}$; its LFM term differs from the interval-midpoint value by only $\mu /(2f_{s})=97.7$ Hz at the nominal setting. Requiring the magnitude of that sample-time frequency to remain below $f_{s}/2$ gives

(15) $$\max_{q}\left|\mu \left(t_{q}-T_{f}/2\right)+f_{d}u\left[q\right]\right|<\frac{f_{s}}{2}.$$

Equation (15) is a necessary sampling screen for the modeled sample-time instantaneous frequency. Up to the stated half-sample LFM term, it also prevents local ambiguity of adjacent principal phase increments. It is not sufficient for reliable recovery under noise, equalization error, cycle slips, fractional delays, or continuous-time pulse shaping. The feasible-region calculation replaces |u[q]| by the exact data-independent bound $u_{\max}=\max_{q}\sum_{k}\left|A_{q,k}\right|$.

Define the lower-triangular cumulative-sum matrix $C\in R^{L\times L}$, with $C_{q,r}=1$ for r≤q and zero otherwise. Equations (9) and (12) give

(16) $$\phi_{c}=Bb,\;B=\kappa CA.$$

Equation (16) linearly relates independent bits to the complete information-phase trajectory. Although the final mapping b↦exp(jBb) is nonlinear, resolving the 2π ambiguity permits regression of the independent bit coordinates over the entire frame.

The generation chain places quadrature/binary coordinates in the DD grid, completes the Hermitian symmetry and DC null, synthesizes the real OTFS drive, accumulates information phase, and adds the centered LFM phase. DD information organization, constant-envelope modulation, and radar sweeping therefore act at the symbol, phase, and sensing layers, respectively. The construction introduces three parameter couplings. DC-row nulling closes the phase but reduces the ideal one-half structural degree of freedom to (N−1)/(2N). Increasing $f_{d}$ separates the phase trajectories associated with different bits but increases instantaneous-frequency excursion and sampling pressure. Increasing $B_{L}$ broadens the basic sweep while reducing the margin available to the information-frequency perturbation in Equation (15). Hence, *N*, *M*, $f_{d}$, $B_{L}$, and $f_{s}$ cannot be selected independently. The actual frame s and phase-generation matrix B are the two interfaces to the receivers: the former supports data-dependent radar matching and complex-waveform equalization, whereas the latter enables framewise phase regression.

## 4. Radar and Communication Receivers

The radar and communication inverse processes are constructed to match the transmit mapping in Section 3. Because the DFRC transmitter knows the current-frame bits, the radar can reconstruct the complete s(b) and evaluate the delay–Doppler statistic

(17) $$\Lambda \left(d,\nu \right)={\left|s^{H}P_{z}^{H}\left(d\right)D^{H}\left(\nu \right)r\right|}^{2}.$$

If only the bare LFM sequence $s_{L}=e^{j\phi_{L}}$ is used as the reference, its normalized coherence with the transmitted common waveform is

(18) $$\eta_{\mathrm{mis}}=\frac{|s_{L}^{H}s|}{{∥s_{L}∥}_{2}{∥s∥}_{2}}.$$

This quantity measures receive–template mismatch; the corresponding matched-filter cell SNR is scaled by $\eta_{\mathrm{mis}}^{2}$. The autocorrelation sidelobes of a data-modulated common waveform remain determined by its actual composite phase.

Unlike the radar, the communication receiver cannot reconstruct a matched reference from known data. With a single synchronized path, removal of the LFM phase would permit adjacent-sample phase differences to estimate u[q]. With multiple paths,

(19) $$y\left[q\right]=\sum_{p}h_{p}s\left[{\left(q-d_{p}\right)}_{L}\right]e^{j2\pi \nu_{p}q/f_{s}}+w\left[q\right].$$

Define the contribution of path *p* as $a_{p}\left[q\right]=h_{p}s\left[{\left(q-d_{p}\right)}_{L}\right]e^{j2\pi \nu_{p}q/f_{s}}$ and the phase-increment operator as $\Delta_{∠}x\left[q\right]=∠\left\{x\left[q\right]x^{*}\left[q-1\right]\right\}$. Even in the absence of noise, this operator is nonlinear and, in general,

(20) $$\Delta_{∠}\;\left(\sum_{p}a_{p}\right)\left[q\right]\neq \sum_{p}\Delta_{∠}a_{p}\left[q\right].$$

Equation (20) shows that phase differencing and multipath summation do not commute. The receiver must therefore recover the complex waveform at the linear propagation layer before inverting the phase-exponential mapping. Dominant-path-aligned differencing serves only as a low-complexity reference.

The complete complex waveform is recovered through the Tikhonov-regularized waveform inverse

(21) $${\hat{s}}_{\mathrm{eq}}=\arg\min_{z}{∥y-Hz∥}_{2}^{2}+\sigma_{n}^{2}{∥z∥}_{2}^{2},$$

whose normal equation is

(22) $$\left(H^{H}H+\sigma_{n}^{2}I\right){\hat{s}}_{\mathrm{eq}}=H^{H}y.$$

All waveforms are normalized to unit average transmitted sample energy, so $\sigma_{n}^{2}$ is dimensionless in Equation (21). Setting the regularization coefficient to $\sigma_{n}^{2}$ provides a noise-level schedule for deterministic stabilization. It is a heuristic choice, not a covariance-derived or Bayes-optimal estimator. The matrix-free implementation forms neither H nor $H^{H}H$: applying H or $H^{H}$ uses *P* cyclic shifts, Doppler rotations, and complex weights, with O(PL) operations per application. Complex conjugate gradients (CGs) solve Equation (22) [45], followed by unit-circle projection,

(23) $${\hat{s}}_{T}=\exp\left\{j∠{\hat{s}}_{\mathrm{eq}}\right\},$$

which discards amplitude variations introduced by linear equalization.

Removing the known LFM phase gives

(24) $$z={\hat{s}}_{T}⊙e^{-j\phi_{L}},$$

and an initial drive estimate is

(25) $$\begin{array}{cc} {\hat{u}}_{\Delta }\left[0\right] & =\frac{1}{\kappa }∠z\left[0\right],\\ {\hat{u}}_{\Delta }\left[q\right] & =\frac{1}{\kappa }∠\left\{z\left[q\right]z^{*}\left[q-1\right]\right\},\;q\geq 1. \end{array}$$

The estimate ${\hat{u}}_{\Delta }$ is inversely mapped to the DD domain and projected onto the quadrature/binary Hermitian set in Equation (4), producing an initial bit vector $b_{0}\in {\left\{-1,+1\right\}}^{K}$. Although this step amplifies adjacent-sample phase noise, it provides a trajectory close enough to the actual phase for reference-aided unwrapping.

Using the initial decision, the complete phase is unwrapped relative to $Bb_{0}$ as

(26) $$\tilde{\phi }=Bb_{0}+\mathrm{unwrap}\left(∠\left[z⊙e^{-jBb_{0}}\right]\right).$$

Tail biting gives Equation (26) a frame-local reference with a deterministic information-phase endpoint. The receiver therefore need not estimate an additional CPM state inherited from the preceding frame, and after LFM removal the wrapped terminal residual ∠z[L−1] should be near zero when equalization and the integer lift are consistent. This endpoint supplies a whole-frame consistency statistic that could be used to reject an inconsistent winding candidate. The evaluated TB-PR receiver uses the closed reference trajectory without thresholding the terminal residual or locating and correcting internal slips; tail biting consequently reduces the state ambiguity presented to unwrapping but does not guarantee slip-free recovery. Equation (26) therefore converts the wrapped sample phases into one continuous frame-level target, and the next step estimates the bit coordinates that best reproduce that target. The framewise regression then solves

(27) $$\overset{˘}{b}=\arg\min_{b\in R^{K}}{∥\tilde{\phi }-Bb∥}_{2}^{2}+\lambda_{\phi }{∥b∥}_{2}^{2},\;\lambda_{\phi }=\gamma \sigma_{n}^{2},\;\gamma >0,$$

with normal equation

(28) $$\left(B^{T}B+\lambda_{\phi }I\right)\overset{˘}{b}=B^{T}\tilde{\phi }.$$

The final decision is

(29) $$\hat{b}=\mathrm{sign}\left(\overset{˘}{b}\right),$$

after which Equation (4) reconstructs the complete DD grid. For $\lambda_{\phi }>0$, $B^{T}B+\lambda_{\phi }I$ is positive-definite, Equation (27) is strictly convex, and Equation (28) has a unique solution. Since the unwrapped error is not strictly complex Gaussian with variance $\sigma_{n}^{2}$, Equation (27) is interpreted as noise-adaptive Tikhonov regression; the experiments use γ=1. Framewise regression alleviates differencing-noise amplification, but an incorrect $b_{0}$ can still cause a cycle slip in Equation (26). The operating region of TB-PR is therefore limited by the reliability of the equalized phase trajectory.

The strong-multipath communication receiver is summarized as follows:

1. Implement matrix-free H and $H^{H}$ from the known path parameters $\left\{h_{p},d_{p},\nu_{p}\right\}$.
2. Solve Equation (22) with complex CG to obtain ${\hat{s}}_{\mathrm{eq}}$.
3. Project onto the unit circle and remove the known LFM phase.
4. Use Equation (25), inverse OTFS mapping, and Hermitian projection to obtain $b_{0}$.
5. Apply the reference-aided framewise unwrapping in Equation (26).
6. Solve Equation (28), hard-decision Equation (29), and reconstruct the DD grid.

The inputs are y, the path parameters, $\sigma_{n}^{2}$, and $\left(N,M,f_{s},f_{d},B_{L}\right)$; the outputs are $\hat{b}$ and the reconstructed grid.

With $I_{\mathrm{CG}}$ iterations, the regularized waveform inverse costs $O(I_{\mathrm{CG}}PL)$ online. The solver uses a zero initial vector, a 120-iteration cap, relative tolerance $10^{-7}$, and γ=1. For fixed $\left(N,M,f_{s},f_{d},\lambda_{\phi }\right)$, dense construction and factorization of $B^{T}B$ cost $O(LK^{2}+K^{3})$ offline, while online phase regression costs $O(LK+K^{2})$. At L=1024 and K=960, the real double-precision phase matrix occupies 7.50 MiB. One full-storage 960×960 normal matrix or Cholesky array occupies 7.03 MiB. Thus, “matrix-free” applies only to channel inversion; dense phase regression remains the principal frame-size limitation. Wall-clock latency was not measured, so real-time feasibility is not claimed.

Reconstructing the current-frame radar reference repeats the Hermitian mapping and separable OTFS transforms, requiring O(LlogL) operations with a fast Fourier transform (FFT) implementation and O(L) phase accumulation and exponentiation. One complex double-precision frame occupies 16 KiB at L=1024. These counts exclude the delay–Doppler search. Hermitian mapping, FFTs, phase accumulation, and reference generation admit streaming or pipelined realization, whereas iterative channel inversion and dense phase regression dominate receiver control and storage. Frame timing, sampling-clock alignment, carrier-frequency correction, and path-parameter acquisition are prerequisites for Equation (21); tail biting is not a synchronization algorithm. Larger grids require operator-form or iterative phase regression because the dense phase matrix and normal factor grow as LK and $K^{2}$, respectively.

Direct evaluation of Equation (17) over $N_{d}$ delay cells and $N_{\nu }$ Doppler cells costs $O(N_{d}N_{\nu }L)$; fast correlation and fast Fourier transforms can reduce this cost on a regular grid. The radar branch considered here is limited to common-waveform range–Doppler matching; angular processing and multitarget association are outside the model.

## 5. Performance Analysis

Waveform, radar, and communication metrics are defined consistently so that the design objectives in Section 2 can be tested directly. The residual imaginary-energy ratio of the unnormalized OTFS synthesis is

(30) ϵim=∥Im{x}∥22∥x∥22,

and the PAPR is

(31) $$\mathrm{PAPR}\left(s\right)=10\log_{10}\frac{\max_{q}{\left|s\left[q\right]\right|}^{2}}{L^{-1}\sum_{q}{\left|s\left[q\right]\right|}^{2}}.$$

Equation (14) ensures samplewise unit modulus in the discrete complex-baseband sequence, and all reported PAPR values use these samples. Pulse shaping, digital-to-analog reconstruction, analog filtering, and radio-frequency nonlinearities can introduce additional envelope variation and are outside the validation model. Occupied bandwidth (OBW) is the narrowest band containing 99% of the discrete power. When the estimate approaches the sampling rate, it is reported as a sampling-limited lower bound; continuous-time spectral behavior then requires a higher sampling rate.

Radar processing is evaluated by correlation structure, detection, and conditional estimation bounds. The ambiguity function is

(32) $$\chi_{s}\left(d,\nu \right)={\left[D\left(\nu \right)P_{z}\left(d\right)s\right]}^{H}s.$$

This definition uses the same zero-padded delay and Doppler sign convention as Equation (17). We report single-frame ambiguity power, cross-frame mean normalized ambiguity power, peak sidelobe ratio (PSLR), and integrated sidelobe ratio (ISLR). Detection probability uses a Swerling-I noncoherent cell model solely to compare matched and mismatched references for the same waveform; for the bare-LFM reference, the cell SNR is multiplied by $\eta_{\mathrm{mis}}^{2}$.

For the conditional local bound, the integer-cell response in Equation (3) is first aligned over the complete sampled frame. Let ${\tilde{t}}_{q}=\left(q-\left(L-1\right)/2\right)/f_{s}$, and let $s_{c}\left(t\right)$ denote a local continuous interpolation of the aligned waveform samples. Referencing the known complex gain α to the frame midpoint absorbs the constant phase associated with the Doppler time origin. The residual-parameter mean and noise model are

$${\left[m\left(\delta \tau ,\delta \nu \right)\right]}_{q}=\alpha s_{c}\left(t_{q}-\delta \tau \right)e^{j2\pi \delta \nu {\tilde{t}}_{q}},\;r=m\left(\delta \tau ,\delta \nu \right)+n_{r},\;n_{r}∼\mathrm{CN}\left(0,\sigma_{r}^{2}I\right).$$

At the aligned point, define waveform derivatives ${\left[d_{\tau }\right]}_{q}=-{\dot{s}}_{c}\left(t_{q}\right)$ and ${\left[d_{\nu }\right]}_{q}=j2\pi {\tilde{t}}_{q}s\left[q\right]$. The corresponding mean derivatives are

$${\left.\frac{\partial m}{\partial \delta \tau }\right|}_{0}=\frac{\partial m}{\partial \tau_{r}}=\alpha d_{\tau },\;{\left.\frac{\partial m}{\partial \delta \nu }\right|}_{0}=\frac{\partial m}{\partial \nu_{r}}=\alpha d_{\nu }.$$

Let $E_{s}={∥s∥}_{2}^{2}$ and let $\rho_{r}={\left|\alpha \right|}^{2}E_{s}/\sigma_{r}^{2}$ be the coherent full-frame energy SNR. The joint Fisher information matrix, including its cross term, is

Jδτδν=2ρrEsRedτHdτdτHdνdνHdτdνHdν≡Jτν.

$$\begin{array}{cc} \mathrm{std}(\hat{\delta \tau }) & \geq \sqrt{{\left[J_{\delta \tau \delta \nu }^{-1}\right]}_{1,1}},\\ \mathrm{std}(\hat{\delta \nu }) & \geq \sqrt{{\left[J_{\delta \tau \delta \nu }^{-1}\right]}_{2,2}},\;J_{\delta \tau \delta \nu }^{-1}\equiv J_{\tau \nu }^{-1}. \end{array}$$

Numerically, ${\dot{s}}_{c}\left(t_{q}\right)$ is evaluated with centered finite differences of spacing $1/f_{s}$ at interior samples and first-order one-sided differences at the endpoints. Thus, the reported curves are conditional local sampled-waveform finite-difference bounds, not exact continuous-time bounds or threshold-performance predictions. They assume known α, exact grid-cell alignment, ideal synchronization, and no clutter or RF impairment.

Communication statistics are evaluated over the independent bits in the positive-Doppler half-plane and Nyquist row of Equation (4). Since the frame energy is $E_{s}=\sum_{q}{\left|s\left[q\right]\right|}^{2}=L$, the energy per independent bit is

(33) $$E_{b}=\frac{L}{K}.$$

Noise is generated with $\sigma_{n}^{2}=E_{b}/10^{{\left(E_{b}/N_{0}\right)}_{\mathrm{dB}}/10}$, where $N_{0}$ denotes the equivalent complex-baseband noise spectral density. All three receivers are compared using the same frame, channel, and noise realization. Each BER point stores both the error count and total tested bits. For a zero-error point, plots use the 95% “rule-of-three” upper bound $3/N_{\mathrm{bit}}$, while tables retain the actual zero count.

Equations (4), (10) and (14) establish real-drive construction, within-frame phase closure, and discrete samplewise unit modulus independently of the channel and signal-to-noise ratio. Radar comparison requires two distinct interpretations. With a fixed transmitted waveform, the difference between the data-dependent and bare-LFM references is governed by Equation (18) and measures whether the receiver uses the known data correctly. Across different transmitted waveforms, ambiguity and CRLB values also depend on actual bandwidth and phase structure and are comparable only under consistent energy, bandwidth, and parameter assumptions. The numerical results therefore report template mismatch separately from conditional estimation bounds. Communication gains likewise have two stages: full multipath waveform recovery mitigates the observed dominant-path BER saturation, whereas framewise regression improves differencing only when the 2π ambiguity can be resolved reliably.

For the feasible-region analysis, let $a_{\mu }$ and $a_{d}$ be multipliers of the nominal LFM slope and CPM phase sensitivity. With fixed frame duration, the LFM bandwidth becomes $a_{\mu }B_{L}$. Since u=Ab and $b_{k}\in \left\{-1,+1\right\}$, the exact data-independent peak bound is $u_{\max}=\max_{q}\sum_{k}\left|A_{q,k}\right|$. The instantaneous-frequency and adjacent-information-phase-increment margins are

$$\begin{array}{cc} m_{f}\left(a_{\mu },a_{d}\right) & =\frac{f_{s}}{2}-\frac{a_{\mu }B_{L}}{2}-a_{d}f_{d}u_{\max},\\ m_{\phi }\left(a_{d}\right) & =\pi -\frac{2\pi a_{d}f_{d}u_{\max}}{f_{s}}. \end{array}$$

Because $u_{\max}$ bounds every legal drive sample, $m_{f}>0$ is a conservative sufficient certificate that Equation (15) holds for all legal bit vectors at the tested parameter pair. If $m_{f}\leq 0$, the certificate is inconclusive rather than a proof of violation because the LFM edge and signed drive peak need not occur at the same sample. Likewise, $m_{\phi }>0$ certifies that every adjacent information-phase increment remains below π in magnitude. The structural degree of freedom $\rho_{H}$ is independent of $a_{\mu }$ and $a_{d}$; the scan uses the engineering threshold $\rho_{H}\geq 0.20$. These sampled certificates neither prove a strictly bandlimited continuous-time waveform nor guarantee a target BER, and they do not define a global joint optimum.

## 6. Numerical Results and Discussion

The numerical study evaluates waveform construction, current-frame radar matching, strong-multipath communication recovery, and parameter tradeoffs using the common metrics in Section 5. Comparisons are restricted to compatible physical quantities and receiver models.

### 6.1. Simulation Setup and Waveform Validation

Table 2 lists the shared waveform, channel, Monte Carlo, and solver settings. Compared methods use identical information, channel, and noise realizations whenever their frame structures permit.

**Figure 3:**
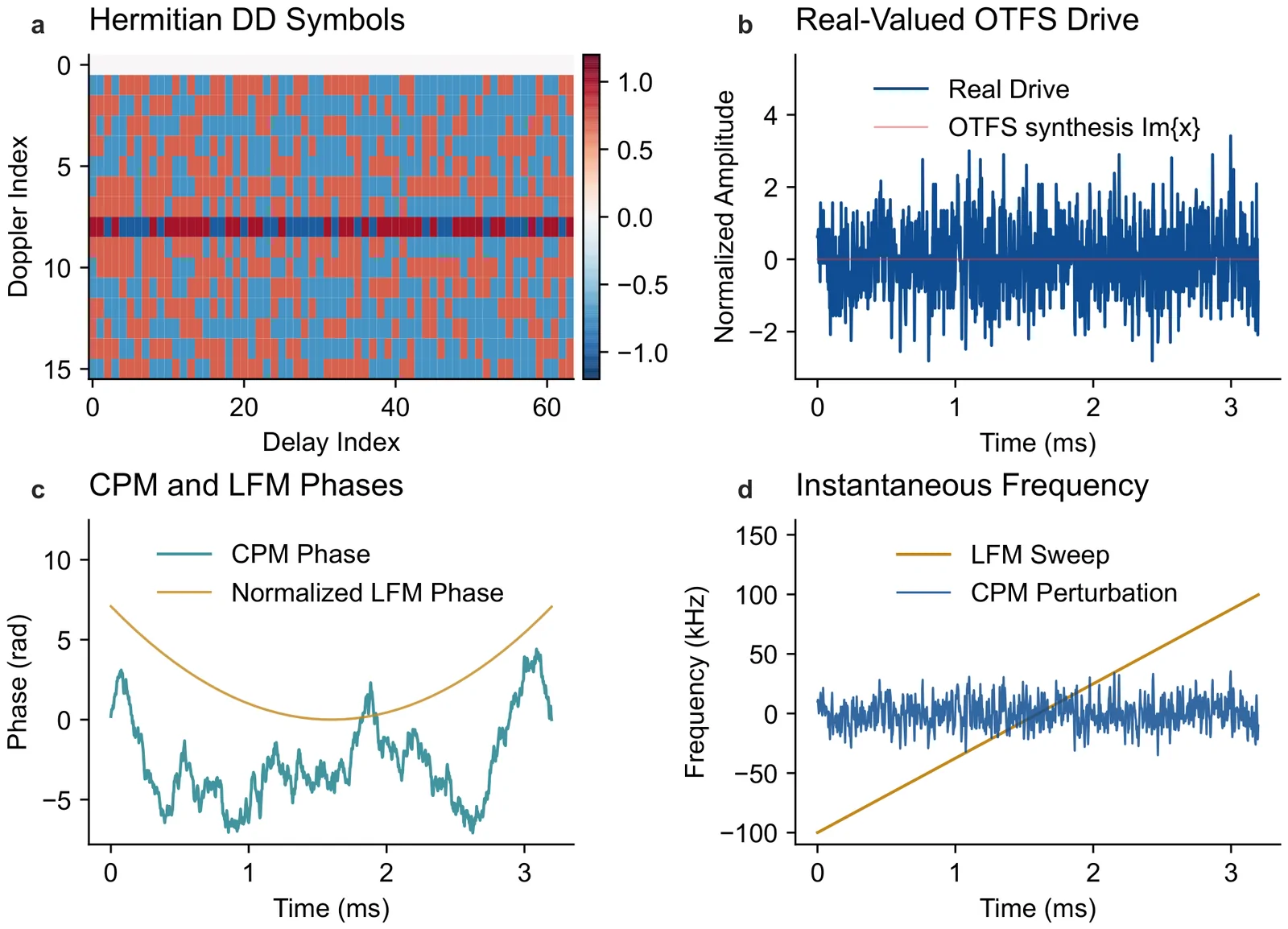
Hermitian DD mapping, tail-biting CPM phase, and composite instantaneous frequency: (**a**) Hermitian DD symbols; (**b**) real-valued OTFS drive and residual imaginary component; (**c**) CPM and normalized LFM phases; (**d**) LFM sweep, CPM perturbation, and composite instantaneous frequency.

Figure 3 verifies the mapping chain. Figure 3b shows the OTFS synthesis Im{x} defined in Equations (7) and (30). The residual imaginary-energy ratio is $1.00\times 10^{-32}$, and the terminal information-phase increment vanishes to numerical precision. These observations confirm the real-drive and zero-sum properties without relying on a selected data pattern.

**Figure 4:**
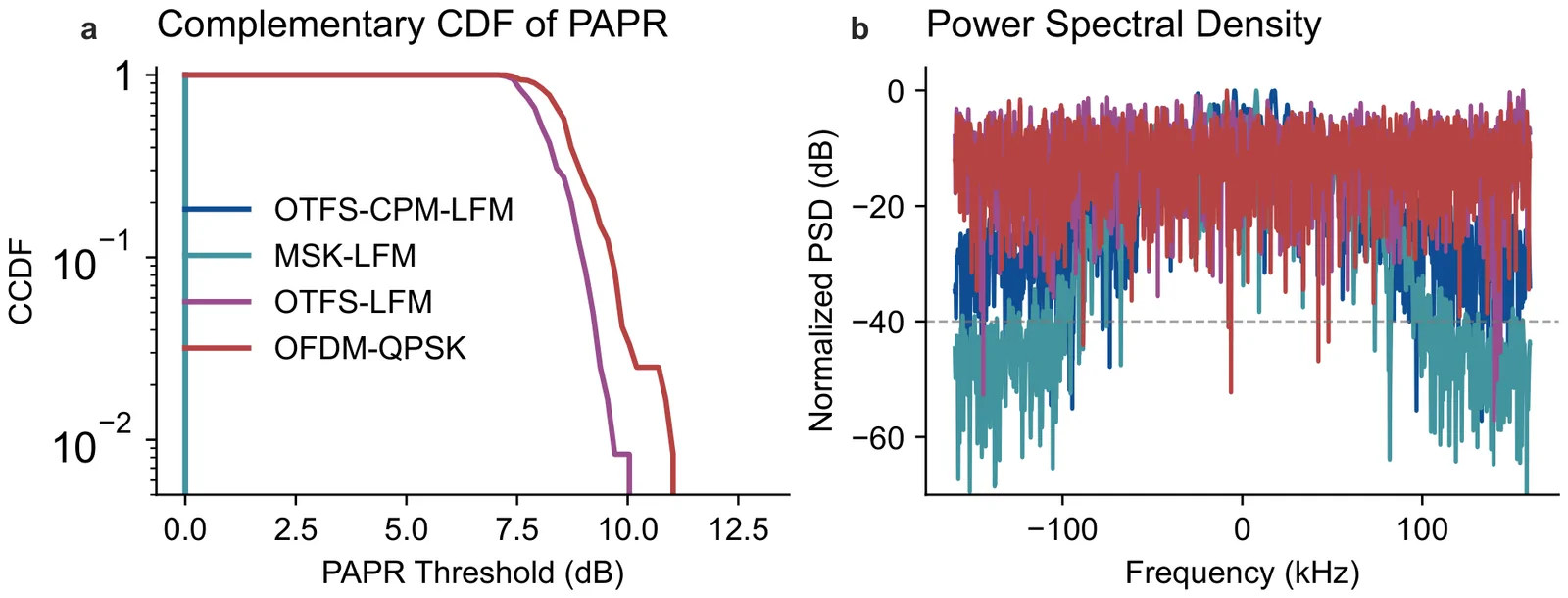
PAPR and spectral characteristics of the compared transmit waveforms: (**a**) complementary cumulative distribution functions of PAPR; (**b**) normalized discrete power spectral densities.

Figure 4 and Table 3 compare envelope and discrete occupied-bandwidth behavior at an equal sample count and unit average energy. The proposed and MSK–LFM samples remain constant modulus, so their PAPR distributions coincide at 0 dB, while the linear references exhibit envelope variation. Spectral estimates near the sampling boundary are reported only as lower bounds. This comparison concerns the sampled transmit waveforms; complete receiver-compatible references are considered in Section 6.4.

The proposed mapping retains $\rho_{H}=0.46875$ of the unconstrained quadrature-grid real coordinates before guard, pilot, and coding overhead. Its constant-modulus result applies to discrete complex-baseband samples and does not imply a constant RF envelope.

### 6.2. Radar Sensing Performance

**Figure 5:**
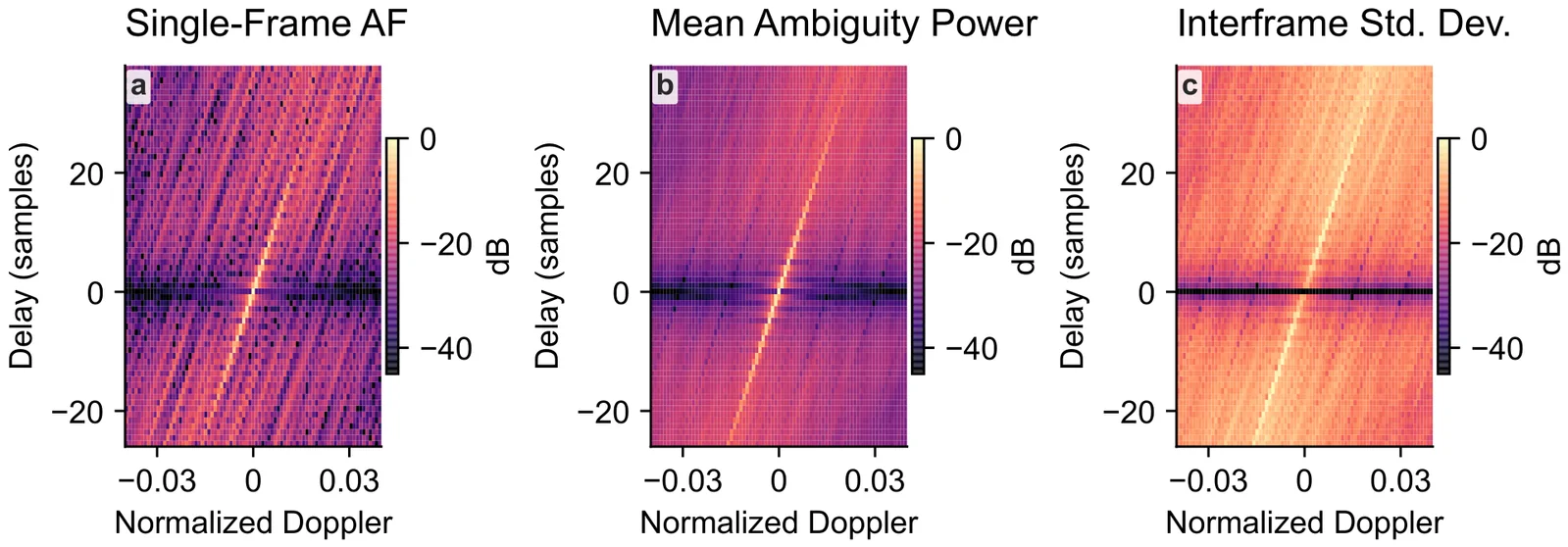
Frame-dependent ambiguity-power statistics of the proposed waveform: (**a**) single-frame ambiguity power; (**b**) cross-frame mean ambiguity power; (**c**) interframe standard deviation of ambiguity power.

Figure 5 separates single-frame ambiguity structure from its cross-frame mean and variability. The data-dependent sidelobe pattern confirms that the radar template must follow the current information-bearing frame.

**Figure 6:**
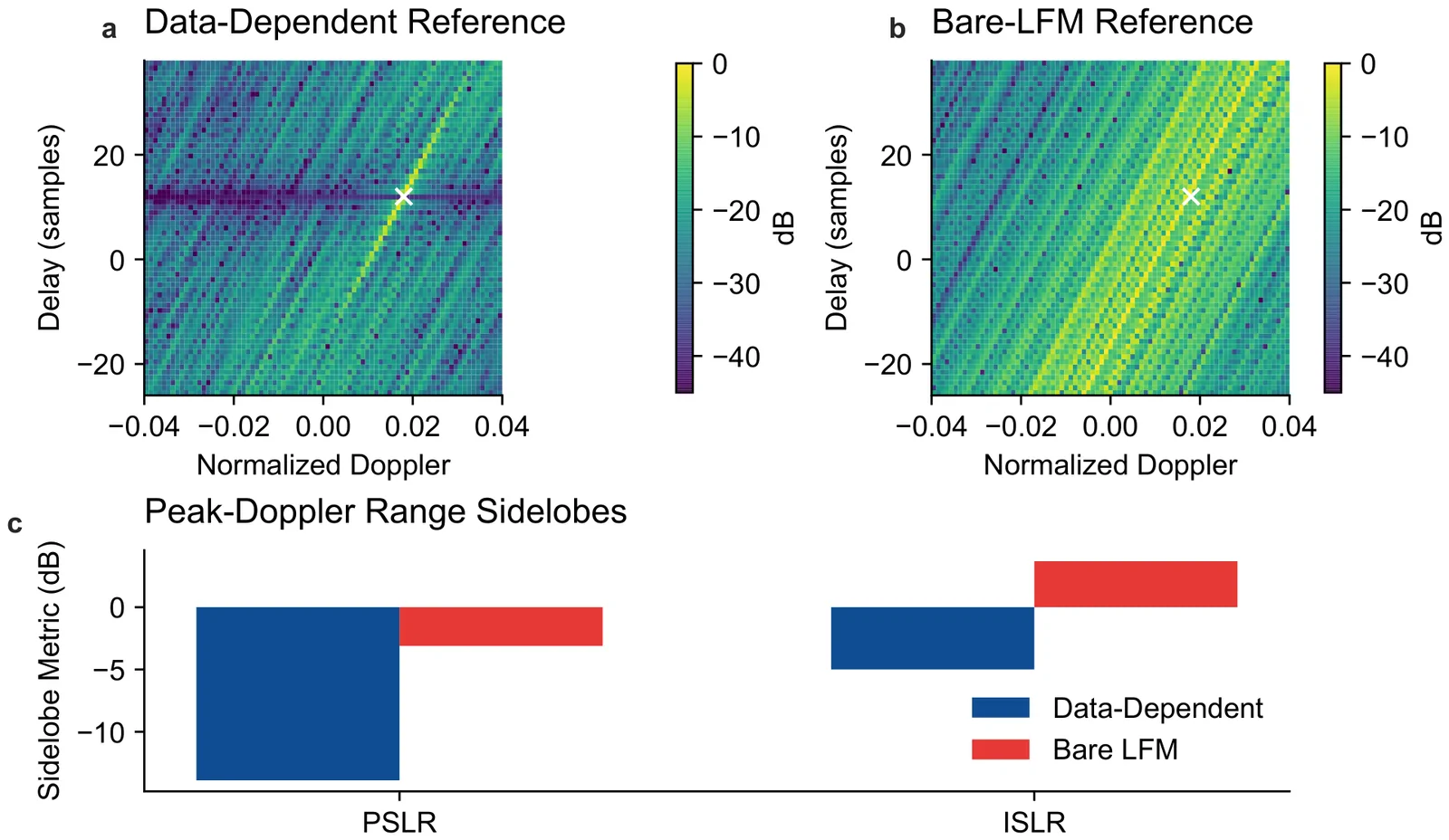
Range–Doppler responses under matched and mismatched radar references: (**a**) response obtained with the data-dependent reference; (**b**) response obtained with the bare-LFM reference; (**c**) peak-Doppler range-sidelobe metrics for the two references.

Figure 6 changes only the receive template. More negative PSLR and ISLR values indicate lower sidelobes. The current-frame reference gives PSLR/ISLR values of −13.90/−4.99 dB. Bare-LFM coherence is 0.189, and its mismatched values degrade to −3.11/3.69 dB. The difference therefore measures template mismatch rather than a change in the transmitted waveform.

**Figure 7:**
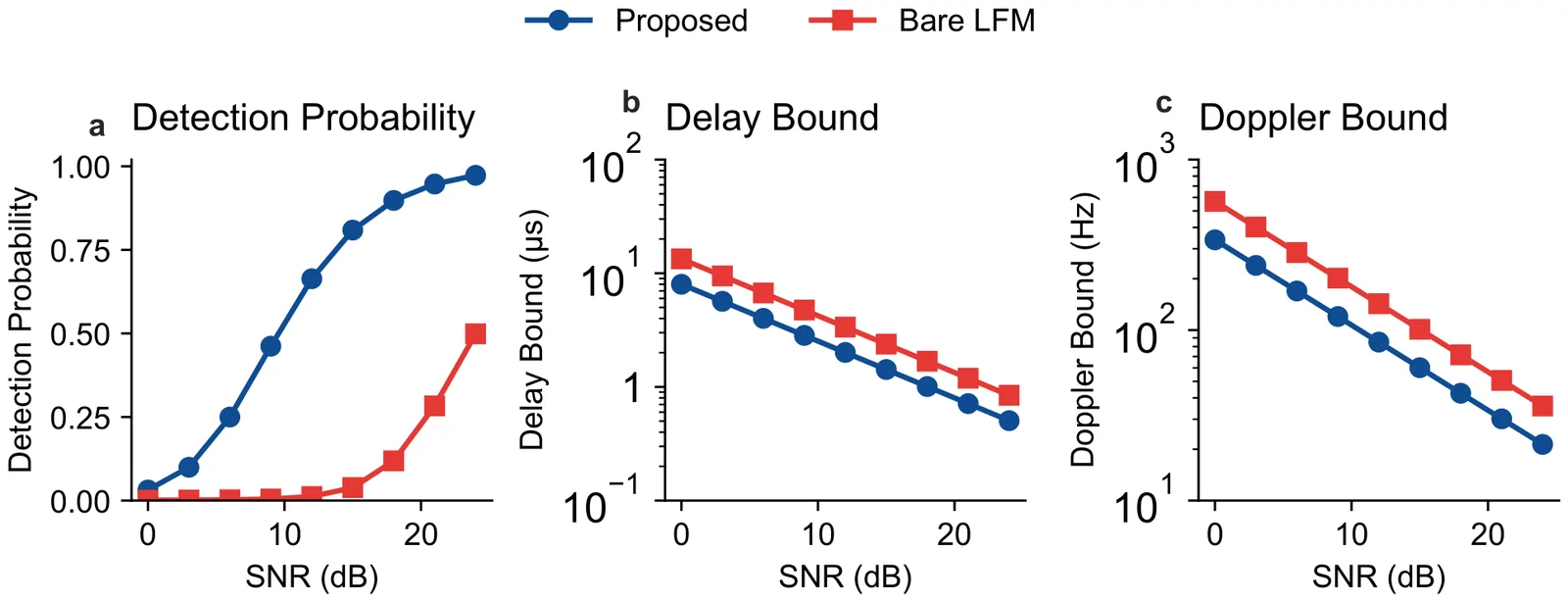
Detection and conditional estimation performance for matched and mismatched references: (**a**) detection probability; (**b**) delay bound; (**c**) Doppler bound.

Figure 7a likewise isolates receive–reference mismatch. At 12 dB SNR, the matched and mismatched detection probabilities are 0.664 and 0.012 for the stated cell model. The proposed and bare-LFM delay bounds are 2.010 and 3.360 μs; their Doppler bounds are 85.0 and 142.8 Hz. These finite-difference bounds are conditional on equal energy, the current data, and known complex gain.

Together, the radar results distinguish the benefit of using the known information phase from intrinsic waveform resolution. They do not represent clutter-limited detection or unknown-gain estimation.

### 6.3. Strong-Multipath Communication Performance

The strong communication channel has complex gains proportional to $[1,0.75e^{j0.7}$, $0.50e^{-j1.0}]$ and is then normalized to unit total power. Its cyclic delays are zero, three, and seven samples, and its Doppler shifts are 800, 920, and 650 Hz. These Dopplers produce multiple phase rotations within one frame. Except for the gain-error study below, the receiver uses perfect CSI. Dominant-path differencing uses only the strongest path, whereas Tikhonov-regularized waveform inversion uses the complete operator.

**Figure 8:**
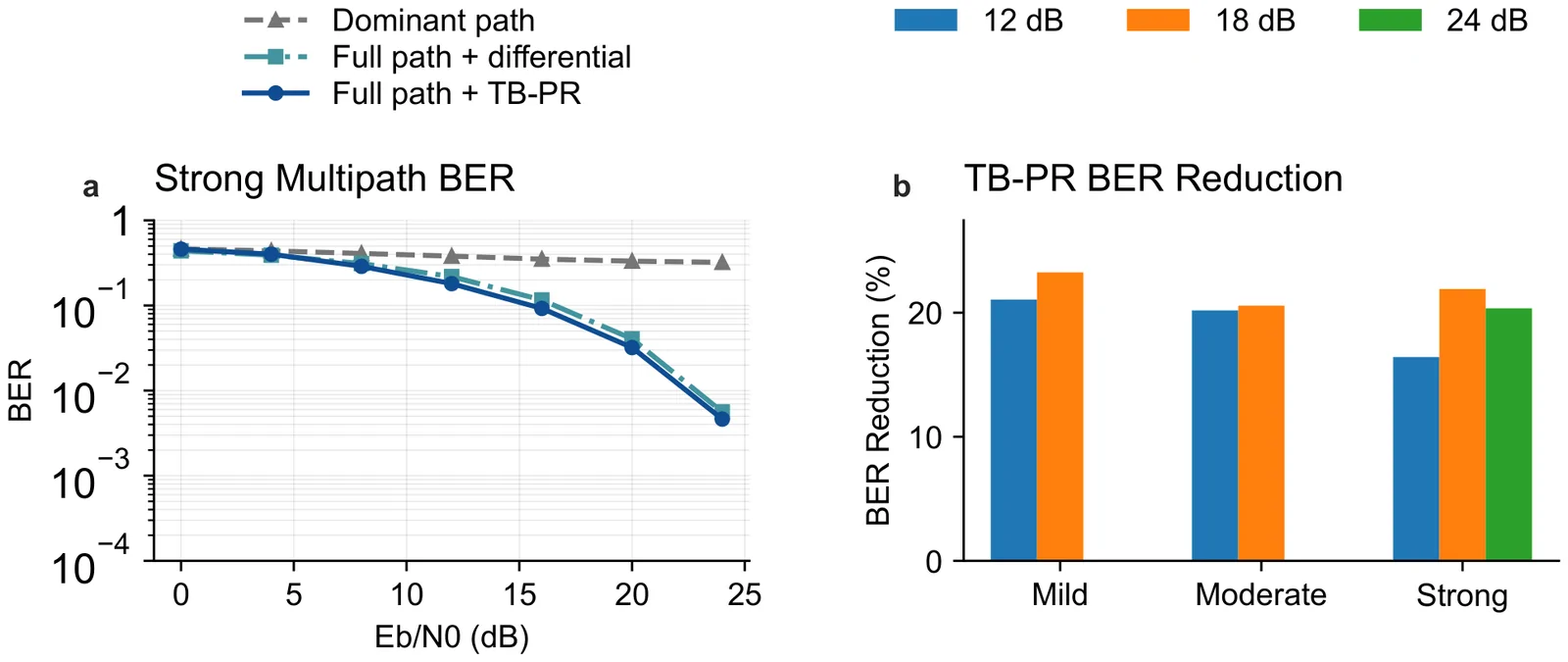
Communication performance under time-varying multipath: (**a**) BER in the strong three-path channel; (**b**) BER reduction achieved by TB-PR across the mild, moderate, and strong multipath profiles.

Figure 8 shows that dominant-path processing saturates under unresolved multipath, whereas full-path inversion continues to improve. TB-PR further reduces BER at the sampled points from 8 to 24 dB. Relative reductions at 12, 20, and 24 dB are 17.3%, 20.9%, and 17.6%, respectively. The mild, moderate, and strong profiles show the same ordering at 18 dB. Points with fewer than 100 baseline errors are omitted from relative-gain curves to avoid overinterpreting finite counts.

The first sampled TB-PR crossover occurs at 8 dB; no threshold between grid points is inferred. At 0 and 4 dB, unreliable preliminary decisions increase phase-cycle slips and make differential detection preferable. This boundary is specific to the tested frame, channel, uncoded receiver, and SNR grid. Candidate extensions include terminal-residual gating, additional phase references, shorter windows, cyclic redundancy check (CRC)- or forward error correction (FEC)-aided candidate selection, and iterative trajectory or channel re-estimation. Their operating-range gains have not been evaluated here.

**Figure 9:**
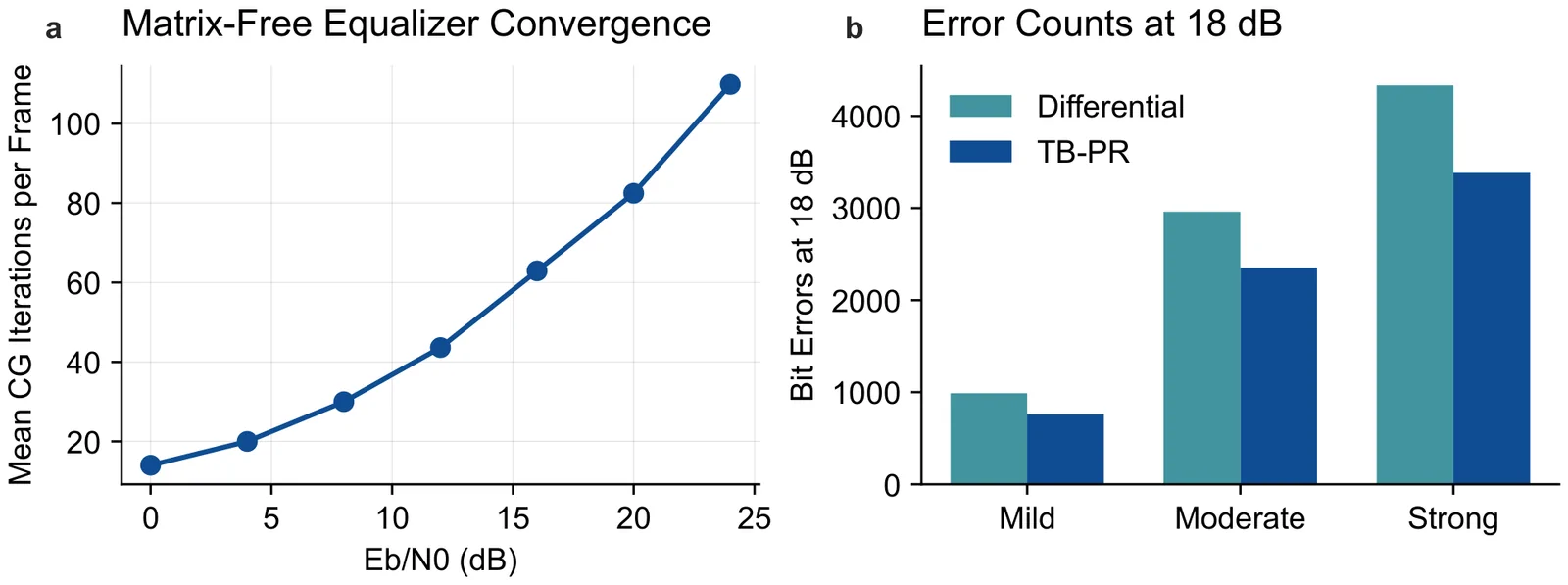
CG solver convergence and receiver error counts: (**a**) mean CG iterations per frame versus $E_{b}/N_{0}$; (**b**) bit-error counts of differential detection and TB-PR at 18 dB across the multipath profiles.

Figure 9 reports the measured solver behavior. At $E_{b}/N_{0}=\left(0,4,8,12,16,20,24\right)$ dB, the mean CG iterations are (14.00,20.00,30.00,43.63,62.93,82.48,109.83). The profile error counts use 57,600 detected bits per receiver. The solver reaches the stated tolerance with convergence in all 120 frames at every point. Lower regularization at higher SNR increases the iteration count. These statistics do not constitute a processing-latency benchmark.

The communication results separate two effects. Full-path inversion mitigates the observed dominant-path saturation, while TB-PR exploits framewise phase structure only after a reliable trajectory has been recovered.

### 6.4. Parameter Sensitivity and Integrated Tradeoffs

**Figure 10:**
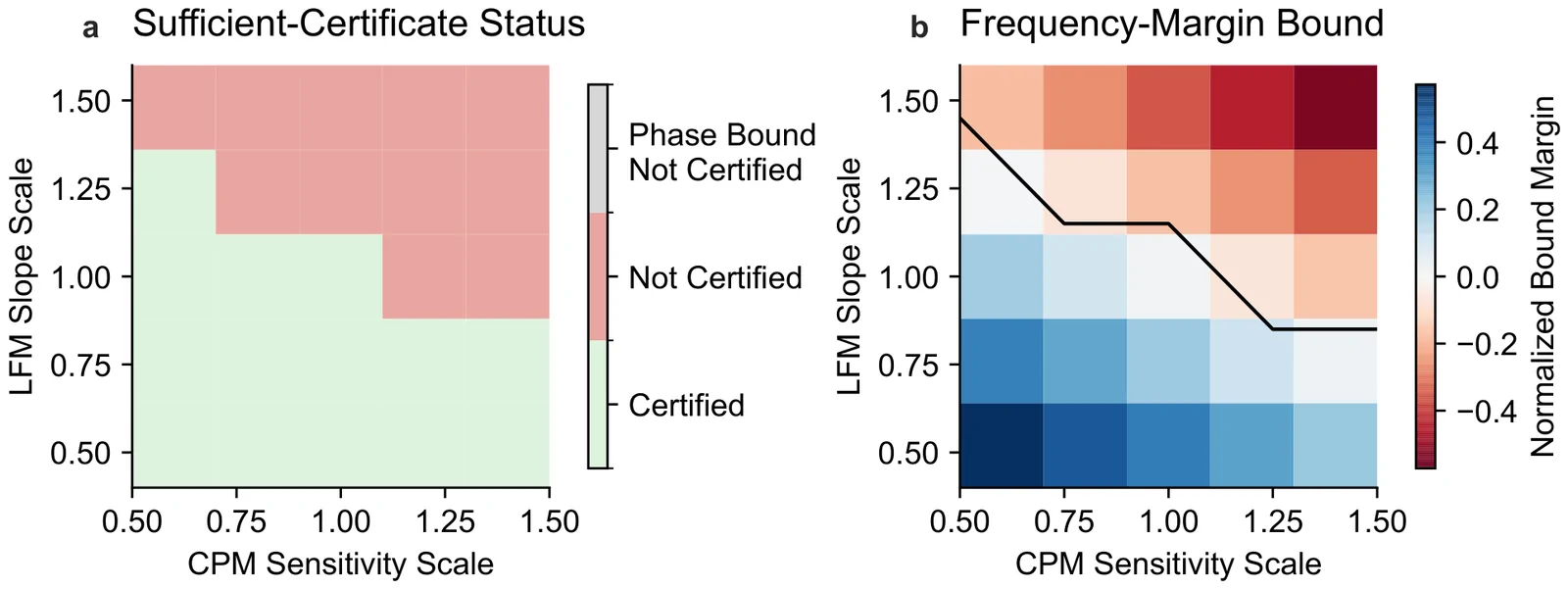
Feasibility certificates over the sampled LFM-slope and CPM-sensitivity grid: (**a**) sufficient-certificate status; (**b**) normalized frequency-margin bound, with the phase-bound status also indicated.

Figure 10 evaluates conservative worst-case certificates for the sampled conditions using the exact legal-frame bound $u_{\max}=\max_{q}\sum_{k}\left|A_{q,k}\right|=3.5645$. The nominal conservative frequency margin is 2.97 kHz. Across the fixed 120-frame waveform evaluation set, the smallest realized sample-time margin is 6.09 kHz. Eight of these frames attain the drive-amplitude bound, but the worst drive and LFM edge need not coincide. The phase-sensitivity scan is $f_{d}/f_{d,0}=0.50:0.25:1.50$, comprising five points for each tested LFM slope. At the largest slope multiplier, none of the five points is certified by both bounds. This finite grid does not establish a continuous feasible boundary or prove violation at uncertified points.

**Figure 11:**
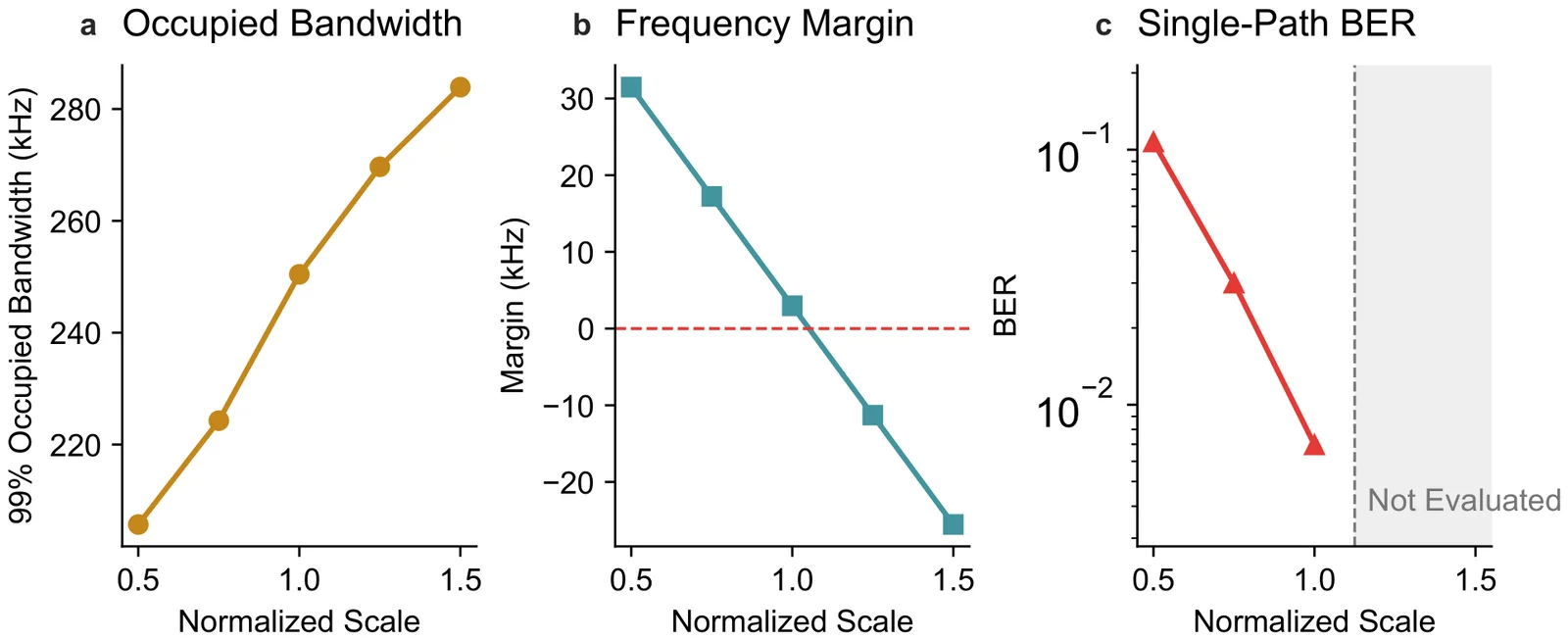
Bandwidth, frequency-margin, and BER sensitivity to CPM phase scaling: (**a**) 99% occupied bandwidth; (**b**) conservative frequency margin, where the red dashed line denotes zero margin; (**c**) single-path BER at the sampled points with positive feasibility certificates.

Figure 11 illustrates the principal design tradeoff. Raising $f_{d}/f_{d,0}$ from 0.50 to 1.00 improves synchronized single-path separation, while the occupied bandwidth broadens and the conservative frequency margin contracts from 31.5 to 2.97 kHz. BER is shown only at sampled points with positive certificates. Unit modulus is preserved because phase sensitivity changes phase rather than sample amplitude.

**Figure 12:**
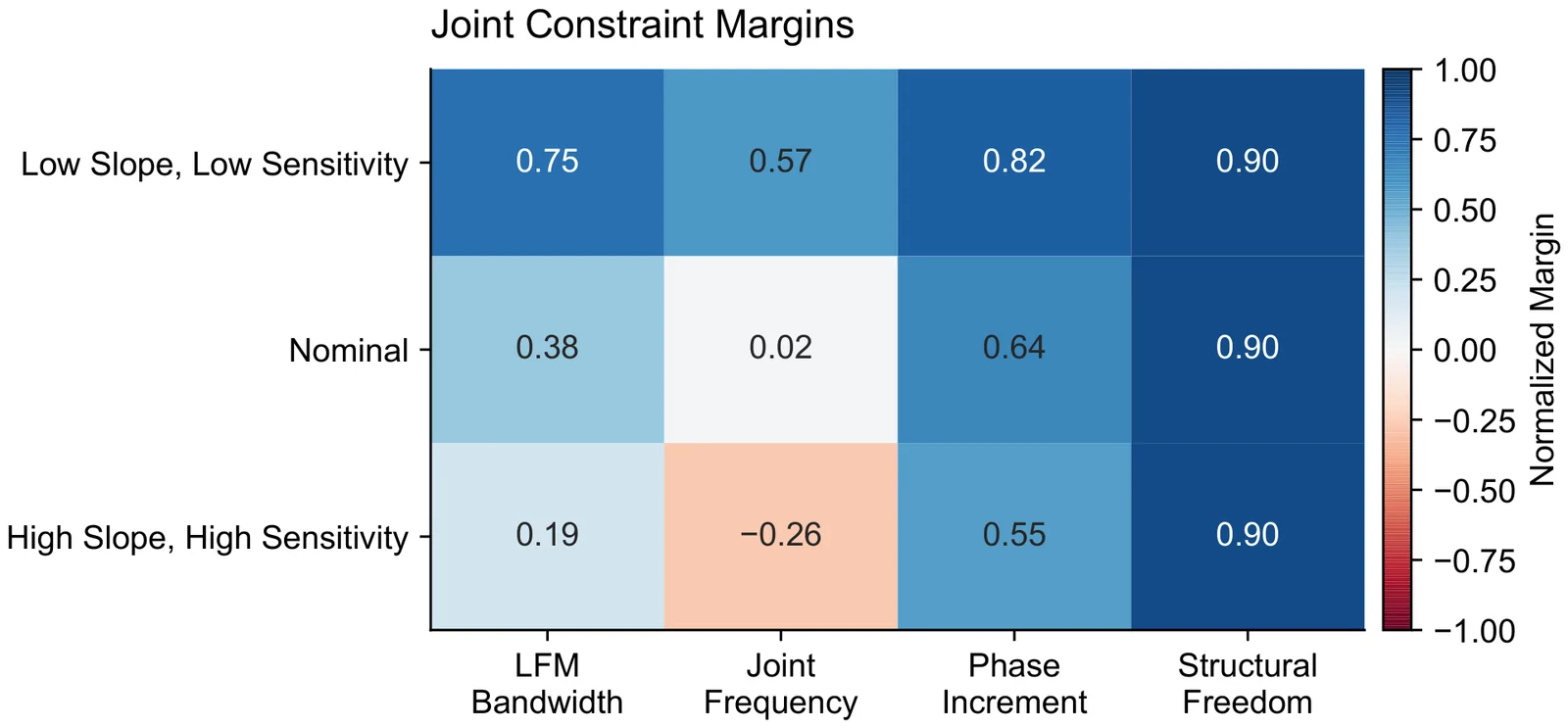
Normalized design-constraint margins at three representative parameter settings.

Figure 12 compares representative constraint balances. The nominal point retains positive structural and frequency certificates, whereas the simultaneous high-slope/high-sensitivity point is not certified by the joint-frequency bound. The analysis therefore characterizes sampled conservative certificates, not a complete feasible set or a global communication–radar optimum.

**Figure 13:**
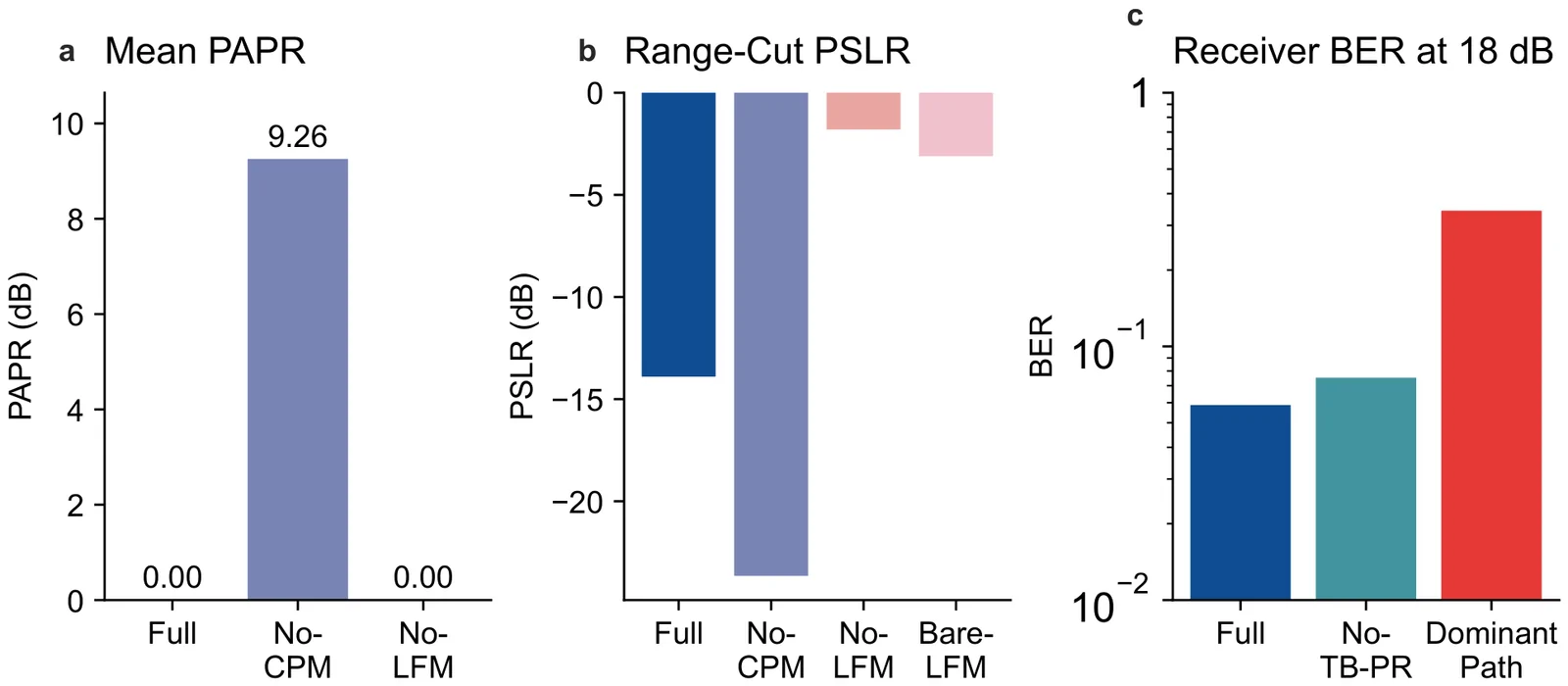
Metric-specific ablation of waveform, radar reference, and receiver processing: (**a**) mean PAPR for the waveform variants; (**b**) range-cut PSLR for the waveform and reference variants; (**c**) receiver BER at 18 dB for the processing variants.

Figure 13 uses a metric-specific protocol, and No-TB-PR denotes post-equalization differential detection. PAPR is evaluated on each transmitted sequence, and PSLR uses the corresponding matched reference except for the deliberate bare-LFM mismatch. No-CPM changes the modulation and requires a different linear receiver; No-LFM tests only the sweep’s range-compression role. BER is therefore not assigned to structurally incompatible variants.

The ablation assigns a distinct role to each component. Hermitian mapping provides the real drive, CPM provides the discrete constant envelope, LFM supplies range compression, and the current-frame reference prevents sensing mismatch. No-TB-PR and dominant-path processing isolate framewise phase recovery and full-path inversion, respectively. These roles cannot be summarized by a single common metric because removing CPM or LFM changes the compatible receiver or sensing objective.

The numerical baseline study uses executable transmitters and compatible receivers with a common sample count, frame duration, sampling rate, sweep bandwidth, average power, channel realization, CSI assumption, and energy-per-bit definition.

**Figure 14:**
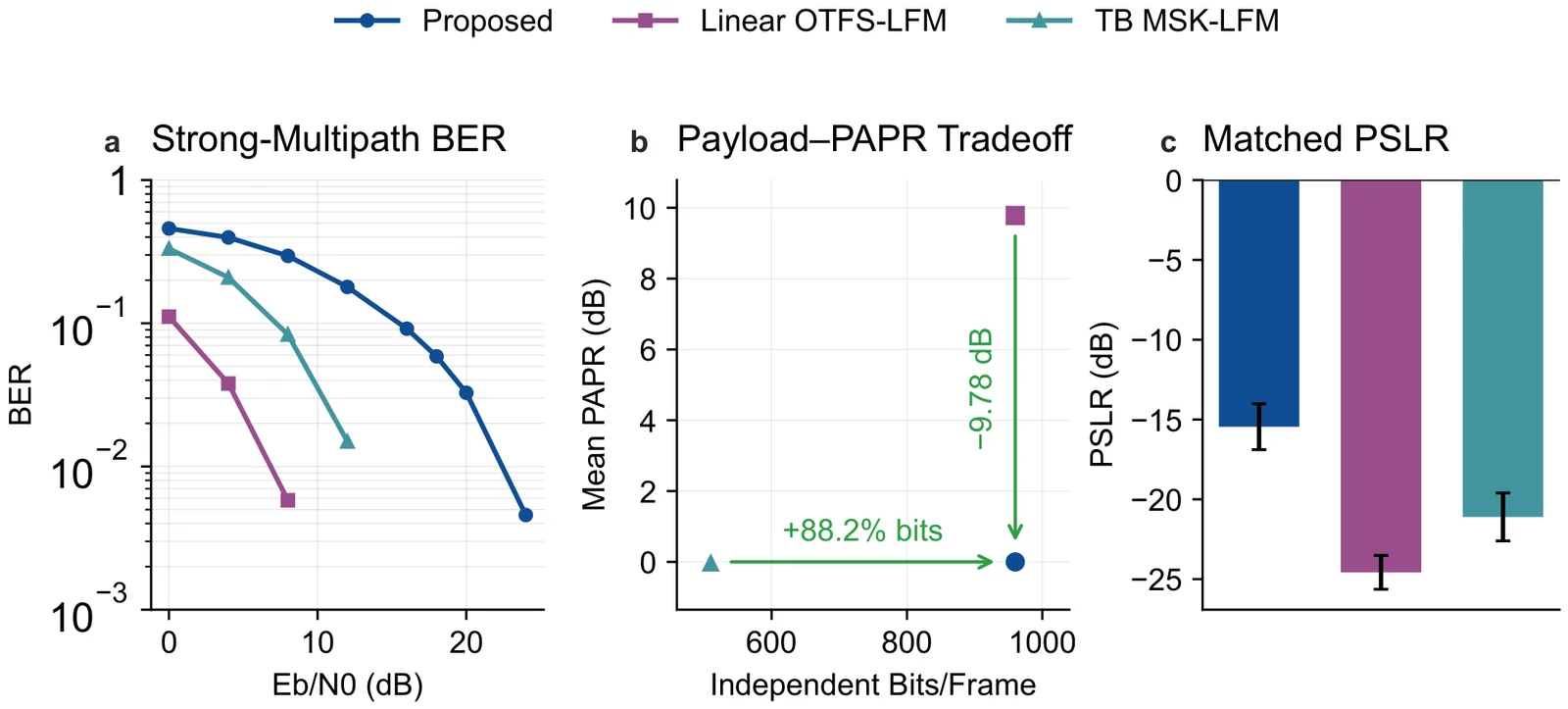
Same-grid comparison of three complete DFRC transceiver chains: (**a**) BER under strong multipath; (**b**) payload–PAPR tradeoff; (**c**) matched-reference PSLR.

Figure 14 compares two complete same-grid references. Linear Hermitian OTFS–LFM retains the 960-bit DD payload but uses a linear envelope. Tail-biting MSK–LFM retains constant envelope with 510 independent bits. Each method uses the common discrete channel and the demodulator appropriate to its transmit mapping.

The proposed receiver has higher uncoded BER and weaker sidelobe suppression than both references under this ideal-CSI configuration. Each BER curve is truncated when its accumulated error count falls below 100. The zero-error linear point is reported through the rule-of-three approximate 95% upper bound, $2.60\times 10^{-5}$, rather than as zero BER. Conversely, the proposed waveform retains the same payload as the linear reference while removing 9.78 dB of discrete-sample PAPR. Under a common peak ceiling and no clipping, this is a 9.78 dB sample-domain peak-headroom relation. It is not a PA drain-efficiency or RF-output result. Pulse shaping, amplitude-to-amplitude (AM/AM) and amplitude-to-phase (AM/PM) conversion, filtering, and spectral regrowth remain unmodeled. Relative to tail-biting MSK–LFM, the proposed method retains constant envelope while carrying the larger frame payload.

Table 4 isolates complex path–gain mismatch. For estimated gains ${\hat{h}}_{p}$, the normalized mean-square error (NMSE) is $\sum_{p}{|{\hat{h}}_{p}-h_{p}|}^{2}/\sum_{p}{\left|h_{p}\right|}^{2}$. Each receiver estimate receives independent circularly symmetric complex Gaussian gain errors, rescaled in each realization to the exact aggregate NMSE. The true propagation, received frames, path delays, and Dopplers remain unchanged. This construction separates gain estimation error from delay or Doppler mismatch without altering the transmitted channel.

The CSI results show progressive BER degradation as gain error increases. The experiment does not test fractional delay, timing or carrier offsets, Doppler-estimation error, or intra-frame channel tracking.

The evidence is bounded by the simulation model. Apart from the gain-error experiment, communication results assume perfect CSI, integer-sample delays, and a cyclic block channel after guard removal. Radar results assume a known current frame, known target gain, and a clutter-free single target. The conditional bounds describe local sampled-waveform information rather than clutter-limited detection. Pulse shaping, DAC reconstruction, analog filtering, oscillator phase noise, nonlinear PA behavior, and RF loopback effects are excluded. The results therefore establish discrete-baseband structure and sensitivity trends, not end-to-end hardware performance.

## 7. Conclusions

This work presents a cyclically closed constant-envelope OTFS–CPM–LFM waveform and a transceiver that follows its physical generation process. The construction links independent DD information to a real zero-sum drive, a closed phase trajectory, and a data-dependent sensing reference. Full-path waveform inversion and framewise phase recovery provide a compatible communication inverse, while current-frame reconstruction prevents radar template mismatch. The results support this integrated architecture but also show that payload, unwrapping reliability, sensing sidelobes, and receiver cost remain coupled. The conclusions are limited to an ideal discrete complex-baseband model and a controlled path–gain uncertainty study. Future work should combine coded slip correction with synchronization and fractional-delay tracking, reduce phase-regression complexity, include nonlinear RF effects, and validate the design on hardware.

## Figures and Tables

**Table 1:** Structural comparison of representative DFRC waveform routes.

Route	Representative Work	Information and Waveform Mechanism	Typical Receiver Model	Main Difference from This Work
OTFS–ISAC	[12,23,24,25]	DD symbols and linear multicarrier synthesis	DD estimation/detection for a linear time-varying channel	No real continuous-phase drive or frame closure
OTFS–chirp	[26]	Composite OTFS–chirp waveform	Chirp/OTFS parameter processing	No Hermitian zero-DC real mapping
LFM–PSK/MSK–LFM	[27,28,29,30]	Phase or continuous-phase embedding in LFM	Matching, dechirping, or symbol demodulation	No DD organization or framewise strong-multipath recovery
Constant-modulus and quantized DFRC	[32,36,37,38,39,41,42,43]	Constant-modulus discrete waveform or array-codeword optimization	Target-matched array or symbol processing	No generative matrix from DD bits to continuous phase
Proposed method	This work	Hermitian DD mapping, zero-DC drive, and CPM–LFM	Regularized waveform inversion and tail-biting phase regression	Joint real drive, deterministic frame closure, and multipath recovery

**Table 2:** Principal simulation parameters and receiver settings.

Parameter	Value	Role
Carrier frequency $f_{c}$	24 GHz	Engineering scenario
DD grid N×M	16×64	Waveform design
Samples per frame *L*	1024	Determined by NM
Independent bits per frame *K*	960	Determined by Equation (5)
Frame duration $T_{f}$	3.20 ms	Waveform design
Sampling rate $f_{s}$	320 kHz	Waveform design
Subcarrier spacing	5 kHz	Determined by *M* and $f_{s}$
Doppler-grid spacing	312.5 Hz	Determined by $T_{f}$
LFM bandwidth $B_{L}$	200 kHz	Engineering scenario
LFM slope μ	62.5 MHz/s	Determined by $B_{L}/T_{f}$
Phase sensitivity $f_{d}$	16 kHz	Waveform design
Frames per main communication point	120	Monte Carlo setting
Frames per multipath profile	60	Monte Carlo setting
Maximum CG iterations	120	Receiver setting
CG initialization and stopping rule	Zero vector; relative tolerance $10^{-7}$	Receiver setting
Regularization multipliers	γ=1; $\lambda_{\phi }\;=\;\sigma_{n}^{2}$	Receiver setting
Random realizations	Shared across compared methods	Common-random-number control

**Table 3:** Discrete-baseband envelope and occupied-bandwidth properties.

Waveform	Mean PAPR (dB)	95th-Percentile PAPR (dB)	Mean 99% OBW (kHz)
OTFS–CPM–LFM	0.000	0.000	249.8
MSK–LFM	0.000	0.000	217.2
OTFS–LFM	8.187	9.177	≥316.5 (sampling limited)
OFDM–QPSK	8.698	9.843	≥316.7 (sampling limited)

**Table 4:** Receiver sensitivity to complex path–gain CSI error at $E_{b}/N_{0}=18$ dB.

Gain NMSE	Differential BER	TB-PR BER
Perfect	0.07479	0.05878
−30 dB	0.07643	0.06030
−20 dB	0.08878	0.07317
−15 dB	0.11760	0.10296
−10 dB	0.17471	0.16379

## Data Availability

The processed data supporting the findings of this study are included in the article. Further inquiries can be directed to the corresponding author.
